# Embouchure dystonia: a video guide to diagnosis and evaluation

**DOI:** 10.1186/s40734-016-0035-x

**Published:** 2016-06-18

**Authors:** Steven J Frucht

**Affiliations:** 5 East 98th Street, New York, NY 10029 USA

**Keywords:** Embouchure, Dystonia, Musician

## Abstract

**Background:**

Embouchure dystonia is an unusual focal task-specific dystonia affecting the muscles that control the flow of air into the mouthpiece of a brass or woodwind instrument. The complexity of the embouchure and the relative rarity of the condition pose barriers for recognition and management of the disorder.

**Methods:**

Case review and video survey.

**Results:**

This paper presents four video compilations that illustrate the rich phenomenology of embouchure dystonia, in order to enhance recognition and diagnosis.

**Conclusion:**

The phenomenology of embouchure dystonia is discussed.

**Electronic supplementary material:**

The online version of this article (doi:10.1186/s40734-016-0035-x) contains supplementary material, which is available to authorized users.

## Background

Embouchure dystonia (ED) is a focal task-specific cranial dystonia affecting the muscles of the lower face, tongue, jaw and pharynx used to control the flow of air into the mouthpiece of a brass or woodwind instrument. In three prior papers, we summarized the clinical phenomenology and natural history of ED [1–3]. ED may affect brass instrumentalists (trumpet, French horn, trombone, tuba) and woodwind players (piccolo, flute, oboe, clarinet, saxophone, bassoon). It typically presents as a painless deterioration in playing, progressing over months to years, and usually ends professional performance. Patients may be categorized by their predominant phenotype: task-specific tremor; lip-pulling; lip-lock; jaw; tongue; and task-specific Meige [2]. Playing difficulty is often limited to one register (pitch range) or to one specific technique, for example articulations (separated notes) or legato (connected notes). About 10 % of ED patients have coincident writer’s cramp, suggesting a possible genetic predisposition to developing focal dystonia. Patients with ED involving the jaw or tongue frequently experience spread of dystonia to other oral tasks such as speaking or eating, and these dystonic features may persist even if they discontinue playing. ED may stem from a central disorder of cortical and subcortical sensorimotor networks that control the lower face, jaw, tongue and pharynx [4]. More recent work using a novel technique for rapid image acquisition demonstrated complex abnormalities in the activation of these muscles in ED patients relative to unaffected professional musicians [5].

**Fig. 1:**
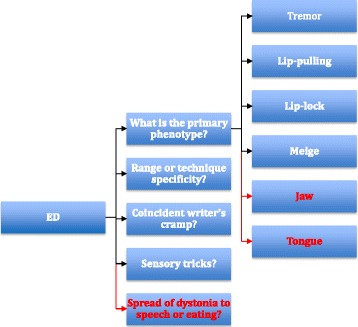
Algorithm for evaluation of ED depicts the thought process behind evaluating patients with ED in the office. The figure proceeds from left to right. Once the diagnosis of ED is suspected (and non-dystonic disorders such as overuse syndrome, inferior orbital neuropathy, embouchure tears and lip trauma are excluded), the first priority is to determine the primary phenotype. Of the six phenotypes, jaw and tongue involvement are noteworthy for their risk of spread of dystonia to speaking and eating (red text). Next, range and technique specificity, coincident writer’s cramp, and the presence of sensory tricks should be ascertained. Finally, if dystonia has spread to speech or eating, or if the jaw or tongue phenotype is present, patients should be counseled to stop playing

The diagnosis of ED remains challenging even for experienced neurologists, and few neurologists are familiar with the mechanics of the embouchure. In this paper, we present a series of video compilations of ED patients to illustrate its rich phenomenology and to facilitate diagnosis (Fig. 1).

## Methods/Consent to publish

Of 109 patients with ED evaluated by the author over a fifteen-year period, 65 available videos were reviewed and categorized. The Mount Sinai Medical Center Institutional Review Board approved the study. All patients shown in videos signed consent allowing publication of their videos in scientific format.

## Results

Thirty-five patient videos were selected and edited into four composite video segments. The first segment (Additional file 1) illustrates practical features for evaluating patients, and the later three (Additional files 2–4) illustrate unique phenomenologic features of ED.

Additional file 1 presents three useful techniques that aid in the evaluation of patients with ED (Additional file 1: Techniques for the evaluation of the embouchure). In addition to carefully observing patients while they play, three approaches are useful when evaluating brass instrumentalists: free buzzing, using an O-ring, and playing using only the mouthpiece of the instrument. “Buzzing” refers to the exercise of vibrating the lips while forming the embouchure. Many brass players warm up before playing by “free buzzing” without a mouthpiece or instrument. Observing a patient’s free buzz can be very informative since the lips are readily visible. The first patient (Free buzz 1) developed task-specific involuntary movements of the upper lip during playing French horn. When he free buzzes, irregular 2–3 Hz anterior protrusions of the upper lip are evident, consistent with lip-pulling dystonia. The next patient, a trombonist, developed loss of control of his embouchure. On free buzzing (Free buzz 2), the upper lip consistently pulls to the right. The next patient, also a trombonist, developed symptoms of ED with prominent tremor. On asking him to free buzz (Free buzz 3), a rapid tremor of the upper lip is visible and audible.

The next three patients illustrate the value of using an O-ring to evaluate brass players with ED. The O-ring is a practice device used by many brass players. It allows the performer to visualize their lips when they buzz: the lips are ordinarily hidden by the mouthpiece during instrumental performance. The first patient (O-ring 1), a trombonist with mild ED interfering with his ability to articulate notes, demonstrates the use of an O-ring. His lips vibrate normally, but there is a slight delay in vibration on starting notes. Legato or connected playing poses no difficulty. The next patient (O-ring 2) developed the tremor variant of ED on tuba. When he buzzes using the O-ring, a rapid tremor of the upper and lower lips is visible and audible. The third patient (O-ring 3) developed the lip-lock pattern of ED, in which his lips come together forcefully when he tries to initiate attacks. He is able to sustain legato playing with the O-ring without difficulty, however when he articulates individual separated notes a delay in initiation of sound is evident.

The final video segment of Additional file 1 ('Techniques for the evaluation of the embouchure') illustrates three patients using just their mouthpiece to play. The first patient (Mouthpiece 1) demonstrates a fast tremor of the upper and lower lips when he blows on his French horn mouthpiece. The next patient (Mouthpiece 2, identical to Free buzz 2), demonstrates a similar pattern using his trombone mouthpiece. The final patient (Mouthpiece 3, identical to Free Buzz 3), demonstrates a similar tremor with sustained notes in the middle register.

By definition, ED is a task-specific form of focal dystonia. Careful evaluation reveals that in many patients with ED, task-specificity is even further refined. In Additional file 2, we explore four aspects of “refined” task-specificity: register-specificity (dystonia occurring in only one pitch range on the instrument); technique-specificity (dystonia triggered selectively by sustained notes or by separated notes); speed (dystonia triggered by specific tempos); and instrument (dystonia triggered by playing one instrument but not by playing others) (Additional file 2: 'Task-specificity in ED').

The first video segment of Additional file 2 ('Task-specificity in ED') demonstrates four French hornists with register-specific ED. The first patient, (Task-specific 1 (TS 1)), develops a fine tremor as he plays a descending scale, affecting notes located within the interval of a perfect fifth. The second patient, TS 2, demonstrates a similar tremor in the same register, with minimal tremor when she plays in the higher registers. The next patient (TS 3) also demonstrates prominent tremor limited to the middle register. The last patient (TS 4) demonstrates inability to sustain airflow and sound only in the highest registers of the horn, a task that she previously accomplished with ease.

The next patient (TS 5) demonstrates technique-specificity in ED. Articulating (i.e., starting notes with a clear beginning) is difficult, producing an unwanted blur to the start of each note. Playing the same notes by slurring (legato playing) presents no problem. The next patient, a tuba player (TS 6, identical to O-ring 3), demonstrates an exquisite speed-dependent trigger for ED. Affected with lip-lock dystonia, his lips come together with excessive force when he articulates notes, producing a delay in the sound. He demonstrates exquisite velocity-dependence of dystonia with normal performance at faster speed, and triggering and worsening of dystonia as his speed slows. The final patient (TS 7) demonstrates exquisite instrument-dependence for ED. Previously able to play flute and saxophone with great fluency, he developed ED of the jaw type, selectively affecting his ability to form the embouchure of the saxophone. Flute playing remains unaffected.

Additional file 3 demonstrates seven ED patients who also possess prominent sensory tricks (Additional file 3: ED—geste maneuvers). Sensory gestes are relatively uncommon in ED, but their effect can be quite dramatic. In some instances, patients were unaware of the trick and the improvement was only discovered during the office evaluation. The first patient (Pt 1) is a trumpeter affected with task-specific Meige, a form of ED where cranial movements are specifically triggered only by playing the trumpet. Touching a particular area at the corner of the right side of his mouth markedly lessens his dystonia. External touch by the examiner to the same location is ineffective, illustrating an exquisite dependence of his dystonia on internal sensory input. The next two patients, both flautists, are affected with ED of the lip-pulling phenotype, with protrusion and forward pursing of the upper lips. In Pt 2, applying mild pressure to the upper lip with the examiner’s fingers or with tongue depressors improves the dystonia, with audible improvement in the quality and volume of sound. Patient 3 displays a similar phenotype, with improvement in the control of airflow when gentle pressure is applied by the examiner to his upper lip.

The next three trombonists (Pts 4–6) developed ED that selectively impaired their ability to produce articulations. All three patients demonstrate improvement with application of a geste. In Pt 4, lip-pulling ED protrudes and pulls his lips apart, blurring his attacks. A gentle touch to the corners of his lips immediately improves his playing, and the improvement even lasts for several seconds after the fingers are removed. Patient 5, afflicted with lip-lock ED, discovered on his own that applying gentle pressure to the corner of the left side of his mouth markedly improves his ability to articulate notes. Similar to Pt 4, this improvement lasts for several seconds after the finger is withdrawn. Pt 6 developed ED affecting the jaw, with inability to maintain a seal on his mouthpiece. Touching the lower jaw, either by the patient or the examiner, improves the dystonia. The final patient, Pt 7, developed ED affecting the jaw related to playing the flute (Additional file 3). Gentle pressure to the lower jaw by the examiner substantially improves the dystonia. She also obtains partial benefit from imagining the sensory geste, a feature seen in some patients with torticollis and blepharospasm [6].

Additional file 4 demonstrates five unusual phenomena in ED (Additional file 4: ED—miscellaneous phenomenology). The first patient (Ph 1, identical to Pt 7 in Additional file 3) developed coincident writer’s cramp twenty years prior to evaluation at our center. Dystonic flexion of the thumb and index finger are improved by holding the pen between her second and third fingers. Mirror dystonia demonstrated as flexion of the right thumb is also evident when she writes with the left hand. The next two patients (Ph 2 and 3), both flautists, developed ED affecting the tongue which first affected their ability to perform rapid tongue articulations on the flute (so called “double and triple tonguing”). By the time of their evaluations, dystonia had spread to involve speech and both had ceased playing. Tongue protrusion and mild movements of the upper and lower lips are triggered by speaking in Patient 2 (Ph 2). She discovered a sensory geste that improved her speech, confirmed in the office by having her hold a plastic syringe between her teeth, either on the left or right side. The next patient (Ph 3) demonstrates moderate dysarthria due to excess tongue tension. A notable improvement in his speech occurs when he employs a sensory trick, holding a plastic pipette between his teeth. He responded very well to treatment with trihexyphenidyl 6 mg daily. The next patient (Ph 4) developed involuntary jaw closure when paying saxophone, which spread over time to occur at rest. On exam, dystonic movements involve the lips, jaw and tongue at rest, but are paradoxically absent when she speaks or reads aloud. The last patient (Ph 5) illustrates the perils of using botulinum toxin to treat ED. Injected with 1.25 units of Botox to the right zygomaticus group, she developed significant right facial weakness which failed to improve her playing and even worsened her ability to compensate for the dystonia.

## Conclusion

In this paper, we have presented four video compilations illustrating the rich phenomenology of ED. We close with a practical algorithm for evaluating patients with embouchure dysfunction in the office.

The first question facing the clinician is to distinguish ED from other embouchure disorders such as overuse syndrome, inferior orbital neuropathy, Satchmo syndrome and mechanical trauma to the lip (these have been extensively reviewed [3]). Once the clinician determines that a patient has ED, the primary phenotype should be defined, and range and technique-specificity should be assessed. The presence of coincident dystonia or sensory tricks should also be sought. Spread of dystonia to speech or eating, or the presence of ED affecting the jaw or tongue should lead to a firm recommendation to cease playing. For other patients, therapeutic approaches may be considered including pedagogic retraining, instrument modification and oral medications such as trihexyphenidyl or propranolol. These approaches are rarely satisfactory, and better treatments are sorely needed for this challenging disorder.

## Additional files

Additional file 1: Techniques for the evaluation of the embouchure Additional file 2: Task-specificity in ED Additional file 3: ED—geste maneuvers Additional file 4: ED—miscellaneous phenomenology

## Abbreviations

ED: Embouchure dystonia
